# Cross-sectional evaluation of bone mass in young adults with Juvenile Onset Systemic Lupus Erythematosus: the role of bone mass determinants in a large cohort of patients

**DOI:** 10.1186/1546-0096-9-S1-P251

**Published:** 2011-09-14

**Authors:** Falcini Fernanda, Stagi Stefano, Cavalli Loredana, Capannini Serena, Masi Laura, Ceri Lorenzo, Denaro Valentina, Matucci Cerinic Marco, Brandi Maria Luisa

**Affiliations:** 1 Department of Internal Medicine, Section of Rheumatology, Transition Clinic, University of Florence, Florence, Italy; 2 Pediatric Unit, Mugello’s Hospital, Borgo San Lorenzo, Florence, Italy; 3 Department of Internal Medicine, Endocrinology Unit, University of Florence, Florence, Italy

## Background

There are few prospective data on bone mass and quality in a large number of patients with Juvenile Systemic Lupus Erythematosus (JSLE). However, there are very few data comparing evaluating the bone mass and quality determinants using dual energy X-ray absorptiometry (DXA), peripheral quantitative computed tomography (pQCT), and quantitative ultrasound (QUS) in JSLE.

## Aim

To assess the prevalence and to identify the main predictors of reduced BMD and bone quality in a cross-sectional evaluation of a large cohort of SLE patients with disease onset before 18 years.

## Methods

Seventy nine patients with juvenile onset SLE (64 females, 15 males, median age 21.0 years, range 14.5 to 39.0 years) treated at the Transition Clinic of Rheumatology Section from January 2008 to December 2010 were evaluated. In all subjects DXA at the lumbar spine, pQCT at radius and phalangeal osteosonography were performed at the same time. The data obtained were compared with 80 age- and sex- matched healthy subjects. All patients at evaluation were receiving Hydroxychloroquine, 50/79 low dose steroids, 35 mycophenolate mofetil, 10 azathioprine and 2/69 had received four infusions of Rituximab.

## Results

At DXA examination, all JSLE patients showed a reduced BMD SDS (p <0.001), total trabecular density, strain strength index, muscle area, cortical bone area, fat area (p <0.001) in comparison to controls. However, we did not find any significant statistical differences regarding AD-SoS and QUS. A significant correlation was found between BMD SDS, disease activity (SLEDAI score), dose and duration of corticosteroids therapy (p <0.001).

## Conclusions

JSLE patients have a low bone mass and impaired bone structure in comparison to controls. A close monitoring of bone mass, a better control of disease activity, physical activity, and a dietary intake of calcium and vitamin D are advocated to ameliorate the bone mass.

